# Prevalence and associated factors of self-reported medical errors and adverse events among operating room nurses in China

**DOI:** 10.3389/fpubh.2022.988134

**Published:** 2022-12-08

**Authors:** Qi Song, Juan Tang, Zhen Wei, Long Sun

**Affiliations:** ^1^Department of Operating Room, Qilu Hospital of Shandong University, Shandong University, Jinan, China; ^2^Center for Health Management and Policy Research, School of Public Health, Cheeloo College of Medicine, Shandong University, Jinan, China; ^3^National Health Commission of China, Key Laboratory of Health Economics and Policy Research, Shandong University, Jinan, China

**Keywords:** self-reported medical errors, self-reported adverse events, prevalence, risk factors, operating room nurse

## Abstract

**Background:**

In recent decades, the prominence of medical errors (MEs) and adverse events (AEs) is fueled by several studies performed across the world. Correspondingly, a high prevalence of medical errors and adverse events have been reported. Operating room nurses (ORNs) were indispensable members of the operating process, and any kind of MEs or AEs from ORNs may cause serious results and even death to the patients. However, to the best of our knowledge, the prevalence and associated factors of MEs and AEs were never reported among ORNs in China, which is the largest country in population and health services quantity in the world.

**Methods:**

This is a cross-sectional study, which was conducted among ORNs in China, and 787 valid questionnaires were analyzed in this study. MEs, AEs, gender, age, married status, religious belief, academic degree, manager or not, working years, working hours/week, physical disease, and mental health were evaluated in this study. MEs were evaluated by eight questions about the occurrence of eight kinds of MEs for the ORNs. For ORNs with MEs, further questions about clinical harm to the patients were interviewed, which analyzed AEs. Kessler 10 was used to evaluate the ORNs' mental health. Logistic regression was conducted to examine the factors associated with MEs and AEs.

**Results:**

The prevalence of MEs and AEs was 27.7 and 13.9% among ORNs, respectively. The most frequent MEs that occurred among ORNs were from surgical instruments (9.1%), disinfection (9.0%), equipment and consumables (8.9%), and specimen management (7.8%). MEs were positively associated with lower working years, poor mental health, and physical disease. The physical disease was positively associated with AEs.

**Conclusion:**

The prevalence of perceived MEs and AEs was at a higher level than other kinds of nurses. Fresh ORNs with physical and mental health problems were the risk population for MEs, and ORNs with physical disease were at a higher risk for AEs. All the findings implied that MEs and AEs were an important issue for ORNs, and ORNs with physical and mental health problems should be paid attention to control MEs and AEs.

## Introduction

Medical errors (MEs) are defined as an incidence when there is an omission or commission in planning or execution that leads or could lead to unintended results (1), and adverse events (AEs) are a type of injury that most frequently is due to an error in medical or surgical treatment rather than the underlying medical condition of the patient (2). In recent decades, the prominence of MEs and AEs is fueled by several studies around the world, which reported a high prevalence of MEs and AEs (3–6). Although both MEs and AEs are inevitable because of imperfect humans (7), controlling MEs and AEs is a fundamental work to protect patient safety.

In the United States (US), MEs were the third leading cause of death (8). However, when we reviewed the studies regarding MEs in China, only a few studies reported the prevalence of MEs among emergency department nurses (9) and medical professionals (10). Regarding AEs in China, most studies focused on the AEs caused by some specific technologies and treatments (11–13). To the best of our knowledge, the prevalence of AEs among medical professionals was less reported in China. As we know, China is the largest country in population and health services quantity in the world (14), and studies about the prevalence and related factors of MEs and AEs were urgent to be conducted in China.

In recent decades, the operation has been one kind of important treatment for patients in the world. Operating room nurses (ORNs), as indispensable members of the operating process, often need to handle urgent situations, such as life-threatening, immediate decision-making. Any kind of MEs from ORNs may cause serious results and even death of the patients (15). However, to the best of our knowledge, the prevalence and associated factors of MEs and AEs were never reported among ORNs in China.

We should know that many previous studies had given us much information about the prevalence of MEs among nurses (16, 17), and many factors were also supported to be associated with MEs for nurses, such as occupational burnout (18), physical and mental health (19), and fatigue (20, 21). However, the differences between ORNs and other kinds of nurses should be considered when we interpret these findings. First, with the high-speed development of operation technologies, ORNs need to learn more new skills than other kinds of nurses. The non-proficient skills in the new technologies may cause them at a higher risk of MEs (22, 23). Second, with the higher concentration on the operating process, ORNs were also at higher risk of physical health than hospitalization nurses (24), which was also a risk factor for MEs (25). Further considering the possible serious outcomes of MEs caused by ORNs, MEs and AEs among ORNs should be explored.

As we introduced before, the prevalence and associated factors of MEs and AEs among ORNs were less reported in China. To fill this gap, we conducted a cross-sectional study among ORNs in China. The main aims of this study were to explore the prevalence and related factors of MEs and AEs among ORNs in China. It not only was helpful for us to learn the prevalence and related factors of MEs and AEs among ORNs in China but also could provide evidence to control MEs and AEs among ORNs in China and some other countries in the world.

## Methods

### Participants and data collection

This is a cross-sectional study conducted among operating room nurses (ORNs) from December 2021 to January 2022 in China. First, the online questionnaire was sent to three nursing supervisors who worked in the operating rooms of three hospitals. Second, they were asked to post the online questionnaire on the different Wechat groups regarding ORNs. Nurses working in the operating room were invited to fill out the online questionnaire anonymously after they agreed on the informed consent. A strict logical check was set for this online questionnaire to avoid logical problems and missing data. Finally, the online questionnaire was clicked 1,256 times during the survey period, and 787 eligible questionnaires were collected in this study with a completion rate of 62.7% (787/1,256).

### Ethics approval and consent to participate

The institutional review board of Shandong University School of Public Health approved the study protocol before data collection. Informed consent was obtained from all participants of the study. All methods were performed in accordance with the Declaration of Helsinki.

### Measures

#### Self-reported medical errors and adverse events

Self-reported medical errors (MEs) were evaluated by eight questions, which investigated the occurrence of eight kinds of MEs for the ORNs in this study. The eight kinds of MEs were about patients or body parts, medication or blood transfusion, specimen management, surgical instruments, equipment and consumables, disinfection, implantable device, and others. For each question, the answers can be chosen from yes or no. In this study, ORNs without any kinds of MEs were coded as without self-reported medical errors (NME), and other ORNs with any positive answers were coded as with self-reported medical errors (YME). For ORNs with MEs, we further asked them if these errors caused clinical harm to the patients, which can be defined as adverse events (AEs) (2). ORNs with MEs responded the negative answers were coded as medical errors without clinical harm (MENH).

#### Social-demographic variables

In this study, the analyzed social-demographic variables included gender, age, married status, religious belief, and academic degree. Gender was coded as male (1) and female (0). Age was analyzed as a continuous variable by the ORNs' years old. Married status was evaluated by one question about the married status for the ORNs. The answers were single, married, divorced, widowed, and others. As there was a small percentage for the last three answers, we recorded it into ever married (1) and never married (0), which the last one contained the last four answers. Religious belief was coded as yes (1) and no (0). The academic degree was evaluated by one question about the highest degree, that the ORNs obtained. The answers were doctors, master, bachelor, junior college, technical secondary school, and others. As most of the ORNs obtained a bachelor's degree, we recorded it as a bachelor's or above (1) and below bachelor's (0).

#### Work-related variables

The work-related variables analyzed in this study contained the professional title, manager, working years, and working hours/week. The professional title was coded as senior (3), medium (2), and junior (1). The manager was evaluated by the question “do you have a management position in their worked hospitals.” Working years were measured by the years they worked in their hospitals. Working hours per week were calculated by two questions about the working day per week and averaged working hours per day.

#### Mental health

Mental health was measured by the Chinese version of the Kessler 10 (K10) scale (26). The Chinese version of K10 was also identified with nice reliability and validity in different populations (27, 28). In the K10 scale, there were 10 items with a 5-point Likert-type response. It was also translated and used to measure mental health and psychological distress in different countries (29, 30). The Cronbach's alpha of K10 in this study was 0.964.

#### Physical disease

The physical disease was evaluated by the question “Were you diagnosed with any kinds of physical diseases?” The answers could be chosen from yes (1) and no (0).

### Statistical analysis

In this study, IBM SPSS Statistics 24.0 (Web Edition) was used to analyze the data. Student *t*-tests or Chi-square tests were performed to compare the differences between ORNs with or without MEs and AEs. Logistic regression was further conducted to examine the factors associated with MEs and AEs. All the tests were two-tailed, and a *p* < 0.05 was considered statistically significant.

## Results

Totally, 787 ORNs finished the questionnaires in this study. Detailed information could be found in the second column of Table 1. In this table, single analyses were also conducted to analyze the factors associated with MEs, and the results supported that the associated factors of MEs were working hours/week (*t* = 2.76, *p* < 0.01), mental health (*t* = 3.35, *p* < 0.001), and physical disease (χ^2^ = 20.91, *p* < 0.001) among ORNs. For the differences between AEs and NMEs, the same factors with MEs were supported. Working hours/week (*t* = 2.58, *p* < 0.01), mental health (*t* = 2.48, *p* < 0.05), and physical disease (χ^2^ = 12.15, *p* < 0.001) were also associated with AEs. However, when we compared the differences between AEs and MENH, all the analyzed factors were not statistically significant (*p* > 0.05). The detailed information is shown in the last three columns of Table 1.

**Table 1:** Sample characteristics and single analyses for the factors associated with MEs and AEs among ORNs [*n* (%)/Mean ± SD].

**Variables**	**Total**	**MEs**			**YME**		***χ^**2**^*** **/** ***t***	
		**YME**	**NME**	***χ^2^*/*t***	**AEs**	**MENH**	**AEs vs. NME**	**AEs vs. MENH**
Observations	787 (100.0)	218 (27.7)	569 (72.3)	–	109 (50.0)	109 (50.0)	109 *vs*. 569	109 *vs*. 109
**Gender**				0.02			0.04	0.03
Male	153 (19.4)	43 (28.1)	110 (71.9)		22 (51.2)	21 (48.8)		
Female	634 (80.6)	175 (27.6)	459 (72.4)		87 (49.7)	88 (50.3)		
Age	33.87 ± 7.07	33.95 ± 6.81	33.84 ± 7.17	−0.20	34.52 ± 6.21	33.38 ± 7.35	−0.93	−1.24
**Married status**							1.42	1.89
Never married	159 (20.2)	42 (26.4)	117 (73.6)		17 (40.5)	25 (59.5)		
Ever married	628 (79.8)	176 (28.0)	452 (72.0)		92 (52.3)	84 (47.7)		
**Religious belief**				0.01			1.17	2.74
Yes	21 (2.7)	6 (28.6)	15 (71.4)		1 (16.7)	5 (83.3)		
No	766 (97.3)	212 (27.7)	554 (72.3)		108 (50.9)	104 (49.1)		
**Academic degree**				2.51			2.99	1.04
Bachelor or above	737 (93.6)	209 (28.4)	528 (71.6)		106 (50.7)	103 (49.3)		
Below bachelor	50 (6.4)	9 (18.0)	41 (82.0)		3 (33.3)	6 (66.7)		
**Professional title**				2.01			4.06	2.75
Junior	387 (49.2)	102 (26.4)	285 (73.6)		45 (44.1)	57 (55.9)		
Medium	362 (46.0)	108 (29.8)	254 (70.2)		60 (55.6)	48 (44.4)		
Senior	38 (4.8)	8 (21.1)	30 (78.9)		4 (50.0)	4 (50.0)		
**Manager**				1.34			1.80	0.64
Yes	69 (8.8)	15 (21.7)	54 (78.3)		6 (40.0)	9 (60.0)		
No	718 (91.2)	203 (28.3)	515 (71.7)		103 (50.7)	100 (49.3)		
Working years	11.36 ± 7.84	11.01 ± 7.53	11.49 ± 7.96	0.76	11.88 ± 7.37	10.15 ± 7.61	−0.48	−1.71
Working hours/week	52.65 ± 11.31	54.45 ± 11.49	51.92 ± 11.17	−2.76^**^	54.97 ± 11.09	53.97 ± 11.09	−2.58^**^	−0.68
Mental health	25.41 ± 8.53	27.05 ± 8.68	24.78 ± 8.39	−3.35^***^	26.95 ± 8.25	27.14 ± 9.12	−2.48^*^	0.16
**Physical disease**				20.91^***^			12.15^***^	0.02
Yes	177 (22.5)	73 (41.2)	104 (58.8)		36 (49.3)	37 (50.7)		
No	610 (77.5)	145 (23.8)	465 (76.2)		72 (49.7)	73 (50.3)		

ORNs, operating room nurses; MEs, medical errors; YME, ORNs with medical errors; NME, ORNs without medical errors; AEs, adverse events; MENH, medical errors without clinical harm; SD, standard deviation.

* p < 0.05.

** p < 0.01.

*** p < 0.001.

The prevalence of different kinds of MEs and AEs is shown in Table 2. In this table, we could find that 27.7% (218/787) of ORNs reported MEs. Among these ORNs with MEs (218 ORNs), 50.0% of ones (109/218) reported the experience of AEs, and the prevalence of AEs among ORNs was 13.9% (109/787). We also analyzed the percentage of different kinds of MEs. The results showed that the prevalence of MEs in patients or body parts, medication or blood transfusion, specimen management, surgical instruments, equipment and consumables, disinfection, implantable device, and others were 2.3, 1.9, 7.8, 9.1, 8.9, 9.0, 3.0, and 0.5%, respectively.

**Table 2:** Prevalence of different kinds of MEs and AEs among ORNs (*n* = 787).

**Kinds of MEs**	***n***	**Percentage**
Any kinds of MEs	218	27.7%
MEs about patients or body parts	18	2.3%
MEs about medication or blood transfusion	15	1.9%
MEs about specimen management	61	7.8%
MEs about surgical instruments	72	9.1%
MEs about equipment and consumables	70	8.9%
MEs about disinfection	71	9.0%
MEs about implantable device	24	3.0%
Other kinds of MEs	4	0.5%
Adverse events	109	13.9%

ORNs, operating room nurses; MEs, medical errors; AEs, adverse events.

In Table 3, logistic regressions were further conducted to analyze the factors associated with MEs and AEs. The results showed that MEs were positively associated with lower working years (OR = 0.94, *p* < 0.01), poor mental health (OR = 1.02, *p* < 0.001), and physical disease (OR = 2.14, *p* < 0.001). When we compared the difference between AEs and NME, only physical disease (OR = 1.89, *p* < 0.01) was positively associated with AEs. However, any analyzed factors were not supported to be associated with MEs (*p* > 0.05), compared with MENH.

**Table 3:** Logistic regressions for the factors associated with medical errors and adverse events among ORNs [OR (95% CI)].

	**MEs**	**AEs vs. NME**	**AEs vs. MENH**
Observations	787	678	218
Male	0.96 (0.63, 1.47)	1.06 (0.61, 1.85)	1.30 (0.62, 2.72)
Age	1.05 (0.99, 1.12)	1.04 (0.96, 1.13)	0.96 (0.86, 1.08)
Ever married	0.98 (0.60, 1.59)	1.12 (0.57, 2.19)	1.22 (0.50, 2.95)
Religious belief	1.18 (0.43, 3.21)	0.34 (0.01, 2.73)	0.13 (0.01, 1.23)
Bachelor or above (Ref.= Below bachelor)	1.61 (0.75, 3.45)	2.32 (0.69, 7.83)	1.77 (0.39, 7.95)
**Professional title (Ref.= Senior)**			
Junior	1.13 (0.39, 3.31)	1.00 (0.25, 4.08)	0.58 (0.07, 5.04)
Medium	1.41 (0.55, 3.64)	1.49 (0.43, 5.15)	0.84 (0.12, 5.94)
Manager	0.78 (0.40, 1.54)	0.57 (0.22, 1.49)	0.36 (0.09, 1.45)
Working years	0.94 (0.90, 0.98)^**^	0.96 (0.91, 1.02)	1.07 (0.98, 1.17)
Working hours/week	1.01 (1.00, 1.03)	1.02 (0.99, 1.04)	1.01 (0.98, 1.03)
Mental health	1.02 (1.00, 1.04)^*^	1.02 (0.99, 1.05)	1.00 (0.96, 1.03)
Physical disease	2.14 (1.46, 3.13)^***^	1.89 (1.16, 3.06)^**^	0.83 (0.44, 1.57)
Constant	0.02^**^	0.01^**^	1.00
*R* ^2^	0.08	0.07	0.08

ORNs, operating room nurses; MEs, medical errors; AEs, adverse events; NME, ORNs without medical errors; MENH, medical errors without clinical harm; OR, odd ratio; CI, confidence interval; Ref., reference.

* p < 0.05.

** p < 0.01.

*** p < 0.001.

## Discussion

In this study, our main aims were to analyze the prevalence and associated factors of MEs and AEs among ORNs in China, and there were several critical findings in this study. First, the prevalence of MEs and AEs was 27.7 and 13.9% among ORNs, respectively. Second, MEs about surgical instruments, disinfection, equipment and consumables, and specimen management were the most frequent errors that occurred among ORNs. Third, factors associated with MEs were lower working years, poor mental health, and physical disease among ORNs. Fourth, the physical disease was positively associated with the occurrence of AEs, and no statistically significant factors were supported between AEs and MENH among ORNs experienced with MEs.

We found that the prevalence of MEs was 27.7% among ORNs. As we know, the reported prevalence of MEs among nurses varied from 37 to 50% in different countries (31–33), which was higher than our findings. The different prevalence of MEs in different countries may be explained by the different healthcare systems and quality (34–36). In China, to our knowledge, this is the first study, which explored the prevalence of MEs among ORNs. However, compared with other kinds of nurses, our findings were similar to the previous findings among emergency department nurses (25.28%) (9) but higher than the findings among the nurses in other departments (17, 37). The higher prevalence of MEs among ORNs and emergency department nurses may be explained by the complicated and urgent work among ORNs and emergency department nurses, which were risk factors for MEs (32, 38).

For the prevalence of AEs among ORNs, we found that 13.9% of ORNs reported the experience of AEs. A study conducted among nursing students found the incidence of AEs was 17.8% (39). In other countries, several studies reported a high prevalence of AEs. In Turkey, a study showed that the prevalence of AEs was 37.3% (40), and the prevalence varied from 51.2 to 63.0% in Iran (41). The main reason for the different prevalence may be caused by the different definitions of AEs in previous studies. In these studies, AEs included all the injury events to patients, and they did not consider if these events were caused by MEs, and MEs were also included in AEs in these studies. The other reason may be caused by the difference between operating room and other departments. Although AEs caused by ORNs may result serious outcomes, the prevalence of AEs may be in lower level. Because nurses in other departments need to serve the patients in a longer time, which is a risk factor for AEs (42).

We also found that lower working years, poor mental health, and physical disease were positively associated with MEs among ORNs. Actually, all these factors were also supported by other kinds of medical professionals in previous studies (43–46), which further reminded us that we should pay attention to fresh ORNs with physical and mental health problems. The reasons may be that ORNs with physical and mental health problems cannot fully engage with patients or the operating process. One of the interesting findings was that working years were not associated with medical errors. Previous supported that burnout was not associated with medical errors (47). Working years, an important factor associated with burnout (48), may also not be associated with medical errors because of the weak association between MEs and burnout. Social-demographic variables were also not associated with MEs, which was also supported in a previous review study (49).

For the associated factors of AEs, we found that physical disease was positively associated with the occurrence of AEs. However, when we compared AEs and MENH among ORNs experienced with MEs, no statistically significant factors were supported. The reasons for the association between physical disease and AEs were similar to the explanation about MEs, which were also supported in other studies (50). The association between social-demographic variables, mental health, and AEs were not supported in this study, it may be caused by the small sample size of ORNs with AEs. Similar reasons also can explain why no factors were supported for the differences between AEs and MENH in this study.

There were also some limitations when we interpret the findings in this study. First, as this is a cross-sectional study, we cannot get any causal relationships between the associated factors and MEs. Second, MEs, AEs, and all the factors analyzed in this study were collected by self-report, and some bias cannot be avoided. For example, some ORNs did not want to report their MEs and AEs (51, 52), and the prevalence of MEs and AEs may be underestimated in this study. Third, the factors analyzed in this study included social-demographic characteristics, worked-related variables, physical disease, and mental health. Although we considered these factors, and there were many factors, which were not analyzed in this study, such as occupational stress (53), patient safety culture (54). In further studies, some other factors should be explored to make a comprehensive understanding of MEs and AEs among ORNs.

## Conclusion

The prevalence of perceived MEs and AEs was at a higher level than other kinds of nurses. Fresh ORNs with physical and mental health problems were the risk population for MEs, and ORNs with the physical disease were at a higher risk for AEs. All the findings implied that MEs and AEs were an important issue for ORNs, and ORNs with physical and mental health problems should be paid attention to control MEs and AEs. Some strategies or policies should be applied to protect the ORNs' health status, which may also play positive roles in patient safety.

## Data availability statement

The raw data supporting the conclusions of this article will be made available by the authors, without undue reservation.

## Ethics statement

The studies involving human participants were reviewed and approved by the Institutional Review Board of School of Public Health, Shandong University. The patients/participants provided their written informed consent to participate in this study.

## Author contributions

QS analyzed the data and drafted the manuscript. JT and ZW collected the data and commented on the draft of this manuscript. LS designed the study and revised the draft. All authors read and approved the final manuscript.
